# The Impact of Maternal Age, Pre-Pregnancy Body Mass Index, Weight Gain and Parity on Glucose Challenge Test (GCT)

**Published:** 2012-03-20

**Authors:** Mitra Arjmandi Far, Saeideh Ziaei, Anoshirvan Kazemnejad

**Affiliations:** Midwifery and Reproductive Health Department, Faculty of Medical Science, Tarbiat Modares University, Tehran, Iran

**Keywords:** BMI, GCT, Maternal Age, Parity, Weight Gain

## Abstract

**Background:**

Gestational diabetes mellitus (GDM) complicates 3-7% of all pregnancies and fetomaternal outcomes are strongly related to early diagnosis of GDM. The aim of this study was to determine the impact of risk factors in the prediction of an abnormal glucose challenge test (GCT).

**Materials and Methods:**

This was a prospective study conducted during 2009-2010 in two prenatal clinics in Rey, Iran. A total of 711 pregnant women who were in their first trimester of pregnancy and met the inclusion criteria were selected. The women were observed once every other week until 24- 28 weeks of gestation. All patients at 24-28 weeks of gestation were screened with 50 g oral glucose GCT. The effects of pre-pregnancy body mass index (BMI), maternal age, and weight gain until the time of GCT, and parity on abnormal GCT were evaluated. All confident intervals were calculated at the 95% level. Data was analyzed using student’s t test and the logistic regression test.

**Results:**

Maternal age (p<0.001), pre-pregnancy BMI (p<0.00), parity (p=0.05) and weight gain during pregnancy (p=0.05), were significantly higher in women with abnormal GCT compared to women who had normal GCT. Logistic regression analyses confirmed that pre-pregnancy BMI (OR=1.09), maternal age (OR=1.14), and weight gain during pregnancy (OR=1.13) were associated with abnormal GCT.

**Conclusion:**

Weight gain had a profound impact on the prevalence of abnormal GCT in our population. Therefore, we propose that pregnant women should only gain the recommended amount of weight during pregnancy.

## Introduction

Gestational diabetes mellitus (GDM) is a common clinical complication that is encountered by obstetricians and their patients. Totally, 3% to 7% of pregnant women suffer from this complication (1). The glucose challenge test (GCT), first introduced by O'Sullivan et al., is a screening test for patients at high risk for GDM (2). Clearly, universal screening is likely to identify most women with GDM. However, the main disadvantage of universal screening is that it requires testing of all pregnant women, most of whom will not have GDM (3). Hackmon and co-workers have reported that mid-trimester maternal body mass index (BMI) and maternal age are useful predictors of abnormal GCT results. They have suggested that these factors should also be considered when selectively screening for GDM (4).

In the search for an alternative, selective approach to this controversial screening method, the impact of different clinical risk factors of age, maternal obesity, ethnicity, and family history of diabetes have been considered in the prediction of abnormal GCT. The aim of this study is to determine whether maternal age, gravidity, BMI before pregnancy, or excessive weight gain during pregnancy are predictive of abnormal GCT results in Iran.

## Materials and Methods

This prospective study was conducted during 2009-2010. On the basis of consecutive recruitment, 711 pregnant women in their first trimester of pregnancy who referred to two university prenatal clinics in Rey, Iran and met the study inclusion criteria were selected. Our inclusion criteria were: i. maternal age between 18-35 years; ii. first trimester pregnancy; iii. no diabetes mellitus, other endocrine or metabolic disorders, cardiovascular diseases, renal or any chronic diseases; iv. single pregnancies; v. no fetal anomalies; and vi.non-smokers.

Patients were enrolled after signing an informed consent. Initial data that included age, parity, gestational age, weight before pregnancy, and family history of diabetes were recorded. The women were observed once every other week until 24-28 weeks gestation. Height was measured to the nearest 0.5 cm with the subjects standing erect and without shoes. Body weight was measured to the nearest 0.1 kg with each subject wearing indoor clothing.

Gestational age was calculated based on menstrual period and confirmed by an ultrasonographic examination performed before 20 weeks of gestation. We apply universal screening for GDM for all our patients, thus all patients at 24-28 weeks of gestation are screened with a GCT (50 g oral glucose)value ≥140 mg/d one hour after the glucose load is considered abnormal and is followed by a 3-hour, 100 g oral glucose tolerance test. Abnormal GCT is determined based on the National Diabetes Data Group Criteria (4). Procedures were approved by the Ethics Committee at Tarbiat Modares University.

Statistical analysis was performed with the use of the student’s t test, chi-square test, and logistic regression analysis (forward method). P values of <0.05 were considered statistically significant. All confident intervals were calculated at the 95 percent level. SPSS (version 11.5) statistical software was used for data analysis.

## Results

The prevalence of abnormal GCT was 14.3% (102/711). The means of maternal age, BMI before pregnancy, parity and weight gain during pregnancy were significantly in pregnant women with abnormal GCT compared to those with normal GCT (Table 1) (p<0.001, p<0.001, p=0.05and p= 0.05, respectively). According to logistic regression analysis, there was a significant association between maternal age (p<0.001), BMI before pregnancy (p<0.004), weight gain during pregnancy until the time of the GCT test (p<0.01), and abnormal GCT (Table 2).

**Table 1:** Comparison of parameters between patients with normal and abnormal GCT at 24-28 weeks of gestation


Parameters	Normal GCT (n=609)	AbnormalGCT (n=102)	P value

Maternal age* (years)mean ± SD	24.81 ± 4.37	27.81 ± 4.11	<0.001
Parity**N (%)			0.05
**Nulliparous**	307 (50.42)	42 (41.18)	
**Multiparous**	302 (49.58)	60 (58.82)	
Pre-pregnancy* BMI (kg/m^2^) mean ± SD	24.59 ± 5.87	27.01 ± 4.54	<0.001
Weight gain until the time of GCT* (kg) mean ± SD	6.03 ± 3.03	6.80 ± 3.65	0.05


*Student t test; **Chi-square test.

**Table 2:** The associations of independent variables (maternal age, parity, pregnancy BMI, and weight gain until the time of GCT on) abnormal GCT


Variable	OR	P value

**Maternal age**	1.14 (1.07-1.21)	<0.001
**Pre-pregnancy BMI**	1.09 (1.03-1.15)	0.004
**Weight gain**	1.13 (1.04-1.22)	0.001


No significant association between other independent variables and abnormal GCT.

## Discussion

The prevalence of abnormal GCT in our study is higher than many parts of the world (6-11). A large body of data shows that maternal obesity is a significant risk factor for the development of gestational diabetes (12-15). This is attributed to the pre-existing insulin resistance associated with obesity and the diabetogenic effect of pregnancy secondary to the presence of counter-regulatory hormones.

Our findings showed that pre-pregnancy BMI was significantly associated with abnormal GCT results. We have also found that weight gain during pregnancy until the time of GCT was significantly higher in the patients with abnormal GCT when compared to women who had normal GCT.

Various aspects of maternal weight gain during pregnancy have been investigated by several authors, and different patterns of maternal weight gain throughout pregnancy have been described (16-18). Abrams et al. (18) found that the mean of weight gain was lowest during the first trimester, peaked during the second trimester, and slowed slightly in the latter part of pregnancy. They also reported that specific patterns of maternal weight gain, particularly during the second trimester, were strongly associated with fetal birth weight. The researchers supposed that mid-trimester maternal fat accumulation is mobilized to support fetal growth during the third trimester.

The impact of maternal weight gain at term on the incidence of GDM is controversial (4). Indeed, some researchers could not establish a clear association between these variables. In contrast, Kabiru et al. have concluded that as maternal weight gain increases, the likelihood of GDM also increases (19). In our study, first and second trimester weight gain was associated with abnormal GCT.

The recognized risk factors associated with increased risk for abnormal GCT have been mostly derived from European and American populations (4); there are few studies that have examined risk factors in other populations. Phaloprakarn et al. have reported that the general risk factors for abnormal GCT were equally applicable to Asian women (14). Our current study supported this finding for Iranian women, since age, pre-pregnancy BMI, weight gain during pregnancy and family history were all significant. As there is a high incidence of GDM among certain ethnic groups, like Iran, ethnicity should be included as one of the risk factors that prompt routine screening for GDM.

We have found that the incidence of GDM in an urban Iranian population is high and commonly recognized risk factors such as pre-pregnancy obesity, maternal age, and weight gain during pregnancy are valid for our population.

## Conclusion

It is clear that the weight gain has a profound and worrisome impact on the prevalence of GCT in our population. Thus, it seems prudent that further emphasis be placed on advising pregnant women to stay within the suggested weight gain range during pregnancy.
